# The microRNA-195 - BDNF pathway and cognitive deficits in schizophrenia patients with minimal antipsychotic medication exposure

**DOI:** 10.1038/s41398-021-01240-x

**Published:** 2021-02-08

**Authors:** Shujuan Pan, Wei Feng, Yanli Li, Junchao Huang, Song Chen, Yimin Cui, Baopeng Tian, Shuping Tan, Zhiren Wang, Shangwu Yao, Joshua Chiappelli, Peter Kochunov, Shuo Chen, Fude Yang, Chiang-Shan R. Li, Li Tian, Yunlong Tan, L. Elliot Hong

**Affiliations:** 1 Peking University HuiLongGuan Clinical Medical School, Beijing Huilongguan Hospital, Beijing, China; 2 Department of Pharmacy, Peking University First Hospital, Beijing, China; 3 Maryland Psychiatric Research Center, Department of Psychiatry, University of Maryland School of Medicine, Baltimore, MD USA; 4 Department of Psychiatry, Yale University School of Medicine, New Haven, CT USA; 5 Faculty of Medicine, Department of Physiology, Institute of Biomedicine and Translational Medicine, University of Tartu, Tartu, Estonia

**Keywords:** Schizophrenia, Diagnostic markers

## Abstract

Cognitive impairment is a core characteristic of schizophrenia, but its underlying neural mechanisms remain poorly understood. Reduced brain-derived neurotrophic factor (BDNF), a protein critical for neural plasticity and synaptic signaling, is one of the few molecules consistently associated with cognitive deficits in schizophrenia although the etiological pathway leading to BDNF reduction in schizophrenia is unclear. We examined microRNA-195 (miR-195), a known modulator of BDNF protein expression, as a potential mechanistic component. One-hundred and eighteen first-episode patients with schizophrenia either antipsychotic medication-naïve or within two weeks of antipsychotic medication exposure and forty-seven age- and sex-matched healthy controls were enrolled. MiR-195 and BDNF mRNA and BDNF protein levels in peripheral blood were tested. Cognitive function was assessed using the MATRICS Consensus Cognitive Battery (MCCB). MiR-195 was significantly higher (*p* = 0.01) whereas BDNF mRNA (*p* < 0.001) and protein (*p* = 0.016) levels were significantly lower in patients compared with controls. Higher miR-195 expression was significantly correlated to lower BDNF protein levels in patients (partial *r* = −0.28, *p* = 0.003) and lower BDNF protein levels were significantly associated with poorer overall cognitive performance by MCCB and also in speed of processing, working memory, and attention/vigilance domains composite score (*p* = 0.002–0.004). The subgroup of patients with high miR-195 and low BDNF protein showed the lowest level of cognitive functions, and miR-195 showed significant mediation effects on cognitive functions through BDNF protein. Elevated miR-195 may play a role in regulating BDNF protein expression thereby influencing cognitive impairments in schizophrenia, suggesting that development of cognition enhancing treatment for schizophrenia may consider a micro-RNA based strategy.

## Introduction

Although current medications can treat psychotic symptoms, schizophrenia remains a debilitating illness often due to cognitive deficits for which currently available medications are not effective^1^. Discovering the underlying neurobiology behind these cognitive deficits may provide alternative strategies for supporting next generation treatment research. MicroRNAs (miRNAs, miRs) are a major regulatory force behind human brain evolution, most abundantly present in the prefrontal cortex that is responsible for many higher cognitive functions^2,3^. MiRNAs are vital regulators for brain development, neuronal differentiation, dendritic spine formation, synaptic plasticity, and learning and memory functions^4,5^. Therefore, MiRNAs may provide a new direction to identify the neurobiological mechanisms responsible for cognitive deficits in schizophrenia.

MiRNAs are small non-coding RNAs, typically consisting of ~21 nucleotides that regulate gene expression by post-transcriptional gene silencing^6,7^. MiR-195 belongs to the miR-15/16/195/424/497 miRNA family, which is closely related to cell propagation and apoptosis^8^. It is abundantly produced in the brain tissue and peripheral blood^9,10^ and has been shown to target the expression of the brain-derived growth factor (BDNF) gene^11,12^. This miRNA binds directly to the 3′-untranslated region (UTR) of the BDNF mRNA and inhibits BDNF protein translation^13^. BDNF regulates neuroplasticity, inhibits the apoptosis cascade, and increases the levels of several cellular proteins required for neurogenesis, neuronal proliferation, and survival^14^. Numerous studies have indicated that reduced BDNF level is related to impaired cognitive function in individuals with schizophrenia^15–17^, but these results have been inconsistent^18,19^.

Similarly, findings of miR-195 levels in schizophrenia are also inconsistent. A meta-analysis indicated overall increased miR-195 level in schizophrenia^20^, and one largest sample size study (*n* = 115) so far reported that the miR-195 level was significantly decreased in antipsychotic-treated patients^21^. Findings from one postmortem study have revealed that reduced BDNF was inversely associated with miR-195 levels in the brain of people with schizophrenia (*n* = 20)^11^. But several other studies showed no significant changes in miR-195 levels in patients with schizophrenia compared with normal controls^22,23^. It is highly likely that the inconsistencies in these studies might be influenced by sample size, illness process, and/or antipsychotic medications. As studies indicated the potential role of miR-195 in controlling brain BDNF expression, it is possible that via affecting BDNF level also in the peripheral blood, miR-195 is harmful for cognitive functions of schizophrenia patients. However, this hypothesis has not yet been verified. Hence, a study jointly examining miR-195 and BDNF translation (mRNA → protein), and involving a larger sample size and first-episode patients with minimal to no antipsychotic exposures, is necessary in order to gain better understanding of the role of miR-195 in BDNF production as well as cognitive function in schizophrenia.

## Materials and methods

### Subjects

Patients (*n* = 118) were first-episode patients with schizophrenia, either never exposed to or within 2 weeks of antipsychotic medication exposure. Specifically, they met the diagnostic criteria for schizophrenia according to the Structured Clinical Interview for DSM-IV (SCID) confirmed by two psychiatrists. They were Han Chinese, 18–45 years old, with illness duration ≤3 years and had to be within 2 weeks of antipsychotic medication initiation at the time of blood draw and cognitive testing. Healthy controls (*n* = 47) were frequency-matched on age and sex to the patient group and were recruited from the local community through advertisements. Controls were excluded if they had experienced DSM-IV axis I psychiatric illness. Complete medical history and physical examination were conducted for all participants. Individuals with significant neurological or medical conditions, or substance abuse (except for tobacco) within the previous 6 months were excluded. Among the patients, 39 were medication-naïve, 19 were on a first-generation antipsychotic (haloperidol) combined with a second-generation antipsychotic (either risperidone or olanzapine), and the remaining patients were on the following second-generation antipsychotics: risperidone (*n* = 30), olanzapine (15), aripiprazole (9), paliperidone (5), and quetiapine (1). For patients on antipsychotic medications, the lifetime exposure was calculated as the total cumulative chlorpromazine equivalent (total CPZ). All subjects provided written informed consent. The study was approved by the Institutional Ethical Committee of Beijing Huilongguan Hospital.

### Demographic and clinical measures

Psychopathology was assessed using the Positive and Negative Syndrome Scale (PANSS), which was administered by one of two trained psychiatrists on the day when blood specimens were collected. The intraclass correlation coefficient (ICC) was over 0.80. Demographic and clinical characteristics of the participants are shown in Table 1.

**Table 1:** Demographic characteristics of fist-episode schizophrenia patients and healthy controls.

Demographics	Schizophrenia (*n* = 118)	Controls (*n* = 47)	*t* or *χ*^2^	*p* value
Sex (M/F)	65/53	27/20	0.08	0.78
Age (years)	28.69 (9.14)	29.59 (6.61)	−0.70	0.49
Education (years)	12.63 (3.42)	14.09 (2.41)	−3.07	0.003
PANSS				
PANSS-P	21.89 (5.21)	N/A	N/A	N/A
PANSS-N	17.24 (5.86)	N/A	N/A	N/A
PANSS-G	36.90 (7.05)	N/A	N/A	N/A
PANSS-T	76.17 (12.27)	N/A	N/A	N/A

All data were reported as mean (SD).

*PANSS* Positive and Negative Syndrome Scale, *P* positive symptom score, *N* negative symptom score, *G* general psychopathological symptom score.

### Neurocognition assessment

Cognitive function was assessed using the validated Chinese version of the MATRICS Consensus Cognitive Battery (MCCB)^24–26^. The MCCB contains assessments of seven cognitive domains: Speed of Processing, Attention and Vigilance, Working Memory, Verbal Learning, Visual Learning, Reasoning and Problem Solving, and Social Cognition. Initial scores were transformed to Chinese-normalized T-scores. In this study, cognitive performance was indexed by the MCCB total score. We also explored the seven domain scores if there was a significant finding associated with the MCCB total score.

### Analyzing miRNA expression by reverse transcription qPCR

Whole blood (5 mL) was collected between 7 and 9 a.m. after overnight fasting using PAX gene TM blood RNA tubes (Applied Biosystems, USA). Tubes were shaken vigorously for at least 10 s after sampling and immediately stored at −80 °C. MiRNAs were extracted using Mag-MAX™ magnetic-bead technology using Mag-MAX^TM^ for Stabilized Blood Tubes RNA Isolation Kit (Applied Biosystems, USA) following the manufacturer’s instructions. RNA was quantified using spectrophotometry, and RNA purity was assessed by measuring the optical density ratios obtained at260nm/280 nm and 260 nm/230 nm. Total miRNA samples were stored at −80 °C. TaqMan^TM^ microRNA reverse transcription kit (Applied Biosystems, USA) was used to convert the miRNA to cDNA according to the manufacturer’s protocol (Applied Biosystems, USA). Quantitative polymerase chain reaction (qPCR) was performed using the 7900HT Sequence Detection System (Applied Biosystems, USA) with the following conditions: 95 °C for 20 s, followed by 50 cycles of 95 °C for 3 s and 60 °C for 30 s. MiRNA-195 level was calculated as relative fold-changes(2^–ΔΔCT^) using a synthetic ath-miR-159 as an endogenous control for normalization.

### Analyzing BDNF expression by reverse transcription qPCR

Reverse transcription of mRNA was performed using a high capacity RNA-to-cDNA Kit (Invitrogen, USA) following the manufacturer’s instructions. qPCR was conducted using a SYBR® Select Master Mix (Invitrogen, USA). PCR was performed with the 7900HT Sequence Detection System as follows: 95 °C for 2 min; 40 cycles of 95 °C for 15 s, and 60 °C for 60 s. Dissociation curves were plotted for every reaction to test the specificity of the amplification. The relative gene expression was calculated with the 2^−ΔΔCt^ method and normalized against the glyceraldehyde 3-phosphate dehydrogenase (*GAPDH*) gene. PCR primers were as the followings: *BDNF*: forward primer: TCACACTCCACATCCCGTGAT, reverse primer: TTACTCTGACCAACGCCCAAA, probe: ACCTCCCAGGCCCCGCTCATT; *GAPDH*: forward primer: CACATGGCCTCCAAGGAGTAA, reverse primer: TGAGGGTCTCTCTCTTCCTCTTGT, and probe: CTGGACCACCAGCCCCAGCAAG.

### BDNF ELISA assay

Whole blood (5 mL) was collected between 7 and 9 a.m. after overnight fasting and centrifuged at 5000 rpm for 10 min. Plasma was immediately separated and stored at −80 °C until assayed. Plasma BDNF protein concentrations were calculated with an ELISA kit (Biosensis, USA) in accordance with the manufacturer’s instructions. The concentrations of BDNF were determined using the sandwich enzyme immunoassay method. Intra- and inter-assay variation coefficients were 4% and 7%, respectively.

### Statistical analysis

Demographic and clinical data were analyzed using analysis of variance (ANOVA) for continuous variables and *χ*^2^ test for categorical variables. Mann–Whitney U test or independent sample *t*-test was used to examine differences in miRNA, mRNA, protein expression levels, and MCCB domain scores between groups. The distributions of BDNF mRNA and protein levels were skewed and they were log_10_ transformed to a normal distribution prior to correlation analyses. Partial correlations were performed with age, sex, and education as covariates during the examination of the relationship between biological and neuropsychological variables. Data are presented as mean and standard deviation (mean ± SD). Statistical tests were considered significant at *p* < 0.05; except for correlations with cognitive measures where statistical tests were considered significant after Bonferroni corrections for multiple comparisons at *p* < 0.05/8 (MCCB total and 7 domains) = 0.00625. Multiple linear regressions were used to further explore the relationships among biological measures, and among cognitive measures and biological measures; all measures were centered and age and sex were included in regression analyses. Finally, a mediation analysis was performed used the PROCESS for SPSS to verify microRNA-195 - BDNF pathway effect on cognitive performance, with a 2000 bias-corrected bootstrap sample for significance testing. All statistical tests were two-tailed.

## Results

### Demographics and clinical characteristics

Demographic and clinical data was showed in Table 1. Patients and controls were not statistically different in age and sex ratio, but their education significantly differed from each other (*p* < 0.05).

### Relationship between levels of miR-195 and BDNF

**Fig. 1:**
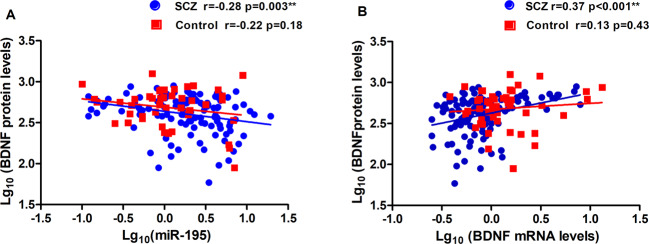
The relationship between miR-195, BDNF mRNA, and protein levels. **A** miR-195 levels were negatively associated with BDNF protein levels in patients with schizophrenia (*p* = 0.003, significant after Bonferroni correction) but not in healthy controls although they were in the same trend. **B** BDNF mRNA levels were significantly and positively associated with BDNF protein levels in patients with schizophrenia (*p* < 0.001) but not in healthy controls. **Statistically significant.

MiR-195 expression levels were significantly upregulated in the peripheral blood of the patients as compared to the controls (*p* = 0.01; Table 2). Patients have significantly lower blood BDNF mRNA (*p* < 0.001) and plasma BDNF protein levels (*p* = 0.016) compared with controls (Table 2). MiR-195 expression was inversely correlated with BDNF protein expression (partial *r* = −0.28, *p* = 0.003; Fig. 1A). BDNF mRNA expression was positively correlated to BDNF protein in the patients (partial *r* = 0.37, *p* < 0.001; Fig. 1B). A significant inverse association between miR-195 and BDNF protein but not BDNF mRNA is consistent with the role of miR-195 in down-regulating BDNF translation However, these correlations were not significant in the controls (all *p* > 0.05).

**Table 2:** MCCB scores, miRNA levels, BDNF mRNA, and protein expression levels.

	Schizophrenia	Controls	*z* or *t*	*p*
MCCB				
Speed of processing	45.08 (8.53)	57.67 (7.76)	−8.42	<0.001
Attention/vigilance	42.27 (8.74)	56.77 (11.22)	−8.47	<0.001
Working memory	46.73 (9.92)	57.86 (5.77)	−8.65	<0.001
Verbal learning and memory	47.95 (11.20)	57.00 (7.37)	−5.89	<0.001
Visual learning and memory	46.28 (9.12)	55.05 (6.38)	−6.75	<0.001
Reasoning and problem solving	48.40 (10.33)	57.14 (6.06)	−6.50	<0.001
Social cognition	47.32 (9.94)	54.26 (10.52)	−3.82	<0.001
Composite score	45.43 (8.69)	58.71 (5.85)	−10.74	<0.001
MiR-195 (2^–ΔΔCT^)	2.89 (2.87)	1.94 (2.13)	−2.59	0.010
BDNF mRNA (2^–ΔΔCT^)	1.20 (1.41)	1.97 (2.46)	4.46	<0.001
BDNF protein (pg/ml)	456.46 (191.13)	545.07 (253.95)	−2.44	0.016

All data was showed as mean (SD).

*MCCB* MATRICS Consensus Cognitive Battery, *BDNF* brain-derived neurotrophic factor.

Multiple linear regression was used to further explore the contributions of miR-195, BDNF mRNA, diagnosis, and their potential interactions to BDNF protein levels (Table 3). MiR-195 (*t* = −2.18, *p* = 0.03) and BDNF mRNA levels (*t* = 4.00, *p* < 0.001) both significantly contributed to BDNF protein, suggesting that the miR195 and BDNF mRNA levels were independently contributing to the BDNF protein levels but in opposite directions. Meanwhile, diagnosis was no longer significantly associated with BDNF protein level, suggesting that the diagnosis effect on BDNF protein level was mediated via miR-195 and BDNF mRNA levels. The effects of age and sex were not significant (all *p* > 0.05).

**Table 3:** Full model regression analysis statistics where BDNF protein level is the dependent variable.

Statistics	Age	Sex	miR-195	BDNF mRNA	Diagnosis	miR195 × diagnosis interaction	BDNF mRNA × diagnosis interaction	miR195 × BDNF mRNA × diagnosis interaction
*t*	0.15	−1.05	−2.18	4.00	−0.09	−0.73	1.59	−0.10
*p*	0.88	0.30	0.03	<0.001	0.93	0.47	0.12	0.92

We considered the possibility that even brief exposure to antipsychotics may have affected miR-195 or BDNF levels. However, miR-195 or BDNF mRNA or protein levels were not significantly correlated with days of exposure (0–13 days) or total CPZ (0–808 mg) (all *p* > 0.05). We also divided the patients into antipsychotic medication-naïve (*n* = 39) and minimal exposure groups (*n* = 79). Comparison showed that miR-195 expression, *BDNF* mRNA and BDNF protein concentrations were not significantly different between them (all *p* > 0.05, Table 4).

**Table 4:** Comparison of miR-195 expression blood BDNF mRNA levels plasma BDNF protein concentrations between medication-free patients and patients exposed in any antipsychotic medications.

	Medication-free (*n* = 39)	Medication-exposed (*n* = 79)	*F* (*P* Value)
miR-195 (2^–ΔΔCT^)	2.70 (2.66)	2.96 (2.99)	0.12 (0.73)
BDNF mRNA (2^–ΔΔCT^)	1.19 (1.20)	1.19 (1.51)	0.002 (0.97)
BDNF protein (pg/ml)	437.97 (188.92)	465.94 (192.77)	0.56 (0.45)

### Relationship between cognition and biological measurements

**Fig. 2:**
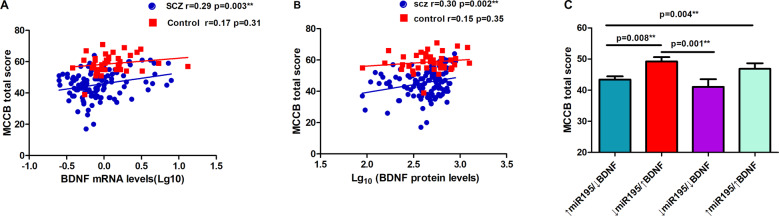
BDNF mRNA levels and cognitive performance. **A** BDNF mRNA levels were significantly and positively associated with MCCB total score in patients with schizophrenia (SCZ) (*p* = 0.003) but not in healthy controls. **B** BDNF protein levels were significantly associated with MCCB total score (*p* = 0.002) in SCZ but not in healthy controls. **C** Patients with ↑miR195/↓BDNF had significantly lower cognitive performance compared to patients with ↓miR195/↑BDNF. **Significant after Bonferroni correction for eight cognitive measures.

Patients had lower MCCB total and domain scores compared with controls (all *p* < 0.001; Table 2). MiR-195 levels were not significantly correlated with MCCB total score or any domain scores (all *p* > 0.05). By contrast, BDNF mRNA levels were significantly associated with the total score (*r* = 0.29, *p* = 0.003; Fig. 2A), and visual learning memory (*r* = 0.27, *p* = 0.005) and speed of processing (*r* = 0.27, *p* = 0.006) domain scores, after Bonferroni correction in the patients. Similarly, BDNF protein levels were associated with MCCB total score (*r* = 0.30, *p* = 0.002; Fig. 2B), and also speed of processing (*r* = 0.29, *p* = 0.002), working memory (*r* = 0.30, *p* = 0.002), and attention/vigilance (*r* = 0.29, *p* = 0.004) domain scores in patients after Bonferroni correction. None of the correlations was significant in the controls although they followed the same trends as in the patients (Fig. 2A, B).

Notably, unlike cognition, clinical symptoms as measured by PANSS total and sub-scores were not significantly correlated with miR-195, BDNF mRNA or protein levels in patients (all *p* > 0.05).

### Joint effects of miR195 and BDNF on cognition

**Fig. 3:**
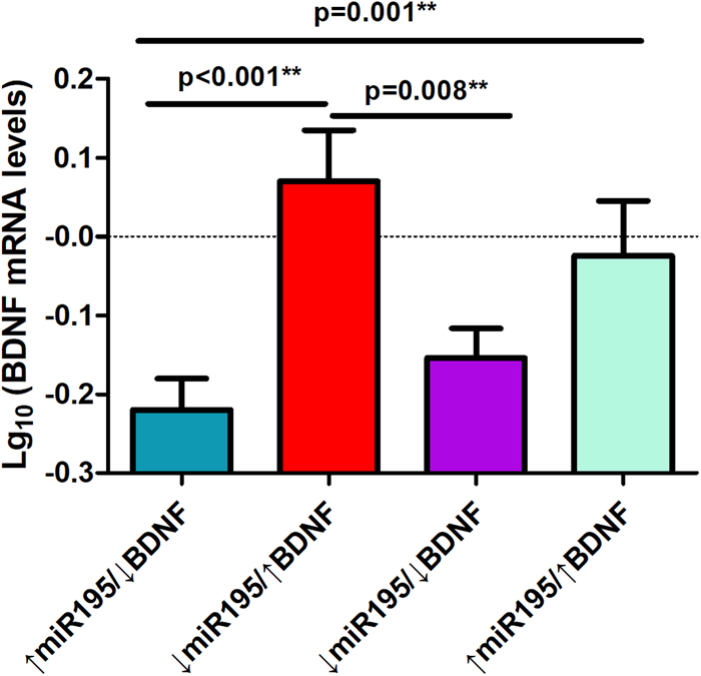
mRNA BDNF level based on median split of miR195 and BDNF protein. Patients with ↓miR195/↑BDNF had significantly higher BDNF mRNA levels compared to patients with ↑miR195/↓BDNF and ↓miR195/↓BDNF.

If our hypothesis is correct, patients with low miR195 and high BDNF (↓miR195/↑BDNF) should have the least cognitive impairment while patients with high miR195 and low BDNF (↑miR195/↓BDNF) should have the most severe cognitive impairment. Accordingly, the patients were divided into 4 groups based on the median of the miR195 and BDNF protein levels. Univariate ANOVA comparing MCCB total and 7 domain scores found that the model was significant for MCCB total score (*p* = 0.004) and working memory (*p* = 0.003), attention/vigilance (*p* = 0.004) after Bonferroni correction. Post-hoc tests showed that patients with ↑miR195/↓BDNF had significantly lower score compared to patients with ↓miR195/↑BDNF for MCCB total score (*p* = 0.008; Fig. 2C), and working memory (*p* = 0.001) and attention/vigilance (*p* = 0.008) domain scores, supporting the hypothesis. Interestingly, patients with ↓miR195/↓BDNF also showed significantly lower scores compared to patients with ↓miR195/↑BDNF for MCCB total score (*p* = 0.001; Fig. 2C), and working memory (*p* = 0.002) and attention/vigilance (*p* = 0.003). This unexpected finding could be due to basal BDNF mRNA levels. We compared BDNF mRNA levels between the two groups and found that BDNF mRNA levels in ↓miR195/↑BDNF groups were significantly higher than the ↓miR195/↓BDNF (*p* = 0.008; Fig. 3), suggesting that the ↓miR195/↑BDNF group may additional have higher mRNA levels to begin with, possibly regulated by other genetic or environmental effects unrelated to the miR-195 levels mechanisms.

### Mediation analyses

**Fig. 4:**
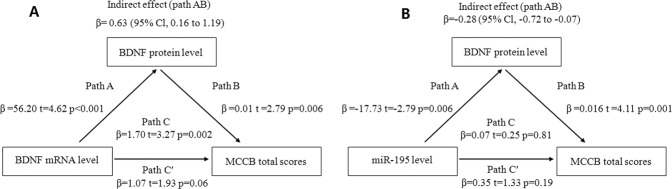
BDNF protein mediated BDNF mRNA and miR-195 on cognitive performances. **A** Analyses were performed to study the mediated effects of BDNF mRNA on MCCB total scores via BDNF protein in the patients. Path AB is the mediation effect and is significant at *P* < 0.05 based on confidence intervals from bias-corrected bootstrapping of 2000 samples. **B** The same analysis using miR-195 as the predictor.

Furthermore, we assessed whether BDNF protein significantly mediated the association between BDNF mRNA or miR-195 and cognitive performance as measured by the MCCB total score. The result showed that BDNF mRNA effects on MCCB total scores were almost entirely mediated by BDNF protein in the patients [*β* = 0.63 (95% Cl, 0.16–1.19)] (Fig. 4A). MiR-195, although no direct effect on MCCB total scores (*β* = 0.07, *t* = 0.25, *P* = 0.81), has a significant effect on MCCB total scores [*β* = −0.28 (95% Cl, −0.72 to −0.07)] through BDNF protein mediation in the patients (Fig. 4B). This is a valid mediation because the opposite signs of miR-195 on BDNF protein versus BDNF protein on cognitive performance^27^. None of the mediation analyses were significant in the controls.

## Discussion

Findings of this study showed that peripheral blood miR-195 concentrations were significantly higher in patients with schizophrenia than in the controls. In this what we believe to be the first report of in vivo relationship between miR-195 and BDNF in schizophrenia, higher miR-195 expression was significantly associated with lower BDNF protein expression in schizophrenia. This is consistent with the reported binding of miR-195 to the 3′-UTR of BDNF mRNA, thus inhibiting BDNF expression^13^. Moreover, BDNF protein levels were found to significantly mediate the effects of BDNF mRNA and miR-195 expressions on cognition in patients with schizophrenia.

Higher plasma miR-195 level in schizophrenia has been reported in previous studies^9,20^. In the only joint study of miR-195 and BDNF of patients with schizophrenia that we are aware of, reduced BDNF was found to be significantly and inversely associated with miR-195 levels in postmortem brains^11^, a finding consistent with our current in vivo study using peripheral blood. The results together imply that miR-195 regulates protein expression of BDNF at the clinical level, and suggest that combined miR-195 and BDNF assessments may represent a useful biomarker reflecting the miR-195 - BDNF pathway abnormalities in schizophrenia.

BDNF mRNA showed strong effects on cognition, but as expected, these effects were largely mediated by BDNF protein. In comparison, miR-195 has no significant direct effect on cognition, which is also expected but miR-195 showed significant mediation effects through BDNF protein on cognition, supporting the hypothesis of the study. Statistically, when there is a strong a priori hypothesis and especially when suppression by the predictor on the mediator is expected as in the case here (i.e., the opposite effect of miR-195 on BDNF protein versus BDNF protein on cognitive performance), a significant mediation is valid even when the direct effect is not significant^27^. Therefore, in schizophrenia patients with minimal to no antipsychotic medication exposures, mediation analyses demonstrated the expected BDNF protein mediation of BDNF mRNA effects on cognition, and importantly the suppressive mediation effect of miR-195 on the positive BDNF protein effect on cognition (Fig. 4B).

However, the underlying mechanisms linking elevated miR-195 and schizophrenia remain unclear. MiR-195 under normal conditions is thought to modulate over-supplied proteins critical to cell cycle, apoptosis, and proliferation, for example *WEE1*, *CDK6*, *Bcl-2*, and *BDNF*, all of which are known target genes of miR-195^28^. As the BDNF protein level is already low in schizophrenia, the elevated miR-195 is unlikely due to a simple feedback mechanism by over-supplies of the BDNF protein. Instead, one possibility is that elevated miR-195 is associated with schizophrenia-related genetic abnormalities, which then suppresses BDNF production. Alternatively, it is possible that the BDNF gene expression abnormality is the primary deficit, leading to reduced BDNF protein levels in schizophrenia. The multiple linear regression and the mediation analyses further suggested a third possibility, that BDNF protein levels were significantly, but only partially, predicted by miR195 levels, because BDNF mRNA levels remained significantly and more strongly contributing to BDNF protein levels, suggesting genetic and/or transcriptional impacts on BDNF mRNA levels, in addition to the miR195 effects, that had influenced BDNF protein levels (Table 4). Animal studies or clinical interventional approaches may be necessary to fully elucidate the causal relationship.

Our current finding of an association between decreased BDNF protein level and multiple reduced cognitive performances in schizophrenia is in line with previous clinical studies done by both ourselves and others^29–31^. It is noteworthy that the generated knowledge in our current study is more advanced as our patients were all at early disease stage with very short or no psychotropic medication history and we addressed more thoroughly the BDNF and its translational regulation. The results and the underlying mechanism we discovered are hence more convincing. Our data are also consistent with the known biological roles of BDNF in regulating glutamatergic transmission, cortical and hippocampal development^32,33^, as well as long-term potentiation that support memory and learning^34–36^. Reduced BDNF concentrations have been reported in the prefrontal cortex and other regions of the brain in schizophrenia and have been speculated to be related to cognitive deficits in schizophrenia^37,38^. Our data showed that miR-195 did not have a direct effect on cognition but it is significantly mediated by BDNF protein on cognitive performance. We may assume that changes occurred in the miR195-BDNF axis in the peripheral blood would also occur with the same direction in the brain, such that BDNF level in patients may also have reduced, hence exacerbating cognition. Meanwhile, it has also been reported that BDNF can pass the blood-brain barrier^39,40^, and changes in the plasma BDNF concentration have been found to be parallel to changes in cerebrospinal fluid^41^. Although miRNA-mediated suppression of BDNF protein concentration is well-established^10,11^, the finding of peripheral BDNF levels influence cognitive impairments in part through peripheral miRNA in schizophrenia is new, suggesting an additional mechanism for the BDNF-related cognitive deficits in schizophrenia.

Interestingly, no significant correlation was observed in control subjects, although the trend was generally the same. This may in part be due to the smaller sample size and thus lower power in the control group.

This study is limited by its cross-sectional design. Longitudinal or interventional designs are needed to ascertain the causal direction between miR-195, levels of BDNF mRNA and protein and cognitive functions. MiR-195 and levels of BDNF mRNA and protein were measured in peripheral blood. If peripheral measures reflect brain concentrations, these findings may suggest that miR-195 could affect cognitive function by regulating the expression of the BDNF gene. However, whether peripheral BDNF levels reflect levels in the CNS in schizophrenia patients remains to be elucidated. Direct miR-195 effects on BDNF protein expression cannot be confirmed with the current clinical study. Finally, we have not investigated and compared the effects of miR-195 with several other miRNAs that may also influence BDNF^42^ and cognition.

To summarize, reduced BDNF levels have long been suggested as a marker for neuroplastic and cognitive deficits in schizophrenia. Our clinical study, consistent with previous postmortem brain study data, suggests that reduced BDNF in schizophrenia may partly be regulated by miR-195. Future development of diagnosis and therapy to correct cognitive deficits through BDNF and other neuroplasticity mechanisms should consider the micro-RNA-BDNF mechanism.
